# Investigating Variation in Compressional Behavior of a Ternary Mixture from a Plastic, Elastic and Brittle Fracture Perspective in the Context of Optimum Composition of a Pharmaceutical Blend

**DOI:** 10.3390/polym15051063

**Published:** 2023-02-21

**Authors:** Hiba Hani Mohammed Ali, Faisal Al-Akayleh, Abdel Hadi Al Jafari, Iyad Rashid

**Affiliations:** 1 Department of Pharmaceutics, College of Pharmacy, University of Sulaimani, Sulaimani P.O. Box 334, Kurdistan Region, Iraq; 2 Department of Pharmaceutics and Pharmaceutical Technology, Faculty of Pharmacy, University of Petra, Amman 11196, Jordan; 3 Department of Pharmaceutical Sciences, Faculty of Pharmacy, Jerash University, Jerash 26150, Jordan; 4 The Jordanian Pharmaceutical Manufacturing Company, Amman 26150, Jordan

**Keywords:** compression, plastic, elastic, brittle, Kawakita, RSM

## Abstract

The choice of optimum composition of a mixture of binary and ternary excipients for optimum compressional properties was investigated in this work. Excipients were chosen based on three types of excipients: plastic, elastic, and brittle fracture. Mixture compositions were selected based on a one-factor experimental design using the response surface methodology technique. Compressive properties comprising Heckel and Kawakita parameters, work of compression, and tablet hardness were measured as the main responses of this design. The one-factor RSM analysis revealed that there exist specific mass fractions that are associated with optimum responses for binary mixtures. Furthermore, the RSM analysis of the ‘mixture’ design type for the three components revealed a region of optimal responses around a specific composition. The foregoing had a mass ratio of 80:15:5 for microcrystalline cellulose: starch: magnesium silicate, respectively. Upon comparison using all RSM data, ternary mixtures were found to perform better in compression and tableting properties than binary mixtures. Finally, the finding of an optimal mixture composition has proven effective in its applicability in the context of the dissolution of model drugs (metronidazole and paracetamol).

## 1. Introduction

The preparation of solid dosage forms involves the use of a number of functional excipients in addition to the active pharmaceutical excipient (API) itself. Ideally, these excipients are generally not blended, either in their native/or unprocessed form(s), or without being subjected to processing under conventional techniques, such as wet/or dry granulation, spray/or freeze drying, spray/or melt granulation, etc. [1]. The aforementioned techniques render powder blends flowable, compressible, and, when compressed, of optimal tablet hardness [2,3]. However, there is a lack of information on how powder blends at different compositions for each type of modified excipient behave upon compression. It is thought that by understanding the optimal composition (type and concentration) of powder blends for excipients in their native/unmodified forms, the limits (concentration) and types (modified or unmodified) of the excipients can be effectively chosen in pharmaceutical powder blends.

In order to elucidate such an understanding, optimum compression is believed to be determined by the overall plastic/elastic and brittle-fracture contributions of a diversity of excipients comprising the whole powder blend. Each excipient undergoes a specific behavior when subjected to compression depending upon its plastic, elastic, both, or brittle-fracture extents [4,5]. However, the mode of compression of a combination of binary excipients, as in the case of pharmaceutical preparations, is relatively different from the compression of individual ones. It has been argued that the overall mode of compression of such a combination is likely to be dependent on the excipient of the strongest property dominance or on the synergistic effects among different modes of compression. In the former, it was suggested that in tablets formulated with binary mixtures of different deformation properties, one material follows the other in its characteristics, and the degree of dependency depends on how strong the property of the dominant material is [6].

The latter has been presented in the improvement of compressibility and compatibility for a solid dosage form comprising elastic and brittle-fracture excipients, Starch and lactose [7], PVP and lactose [8], microcrystalline cellulose (MCC), and silicon dioxide [9] are good examples illustrating such synergistic combinations, despite the fact that not all elastic-brittle excipients show similar synergy. With regard to the foregoing, combinations comprising binary mixtures of plastic materials were coprocessed to enhance optimum tableting with lower-cost novel multifunctional excipients. These types of excipients, which include combinations of alginic acid coprocessed with microcrystalline cellulose, and maize starch coprocessed with pregelatinized starch, are typical excipients of plastic deformation modes [10,11]. Amin and Fell, in addition to Holman and Leuenberger, have illustrated the improvement in tableting properties upon consolidation of different modes of powder blends (plastic/elastic) from a percolation perspective [12,13]. In this regard, they confirmed the existence of percolation thresholds in mixtures containing excipients with plastic deformation/brittle fracture and plastic/plastic deformation properties. Such a threshold was tested using porosity as a key factor in relation to the tablet’s tensile strength.

The work outlined herein, therefore, will elaborate on the understanding of the compression behavior of a ternary mixture composed of three components from a response surface methodology perspective (RSM). Finding an optimum mixture of the mass content and composition will be given great consideration within the context of physically mixed plastic, elastic, and brittle facture excipients. The verification of finding such an optimal mixture composition will be tested on ternary mixtures of different compositions and wet granulation preparation types.

## 2. Material and Methods

### 2.1. Chemicals and Reagents

The following materials, with their suppliers provided herein, were purchased; microcrystalline cellulose PH-101 (Avicel^®^101), lot No. 60997C, particle size 50 µm (FMC, Philadelphia, PA, USA), commercial maize starch (Beijing Quanfeng Starch Company, China), synthetic amorphous magnesium silicate, lot No. 1771 (JAI RADHE, India), pharmaceutical grade metronidazole (Lution-Hong Gou, China), pharmaceutical grade metronidazole (Lution-Hong Gou, China), paracetamol (Sri Krishna Pharmaceutical Ltd., Hyder-abad, India), Flagyl^®^ 500 mg originator product (Sanofi-Aventis), and Panadol^®^ 500 mg originator product (GlaxoSmithKline, Australia).

### 2.2. Preparation of Binary and Ternary Mixtures

MCC, starch, and Mg silicate were blended as binary and ternary mixtures for 10 min using an Erweka cube mixer (KB 155, Langen, Germany) in different ratios, with a total amount equal to 20 g. Then, 10 g of the foregoing was wet-granulated using distilled water. The wet granules were passed through a 2.0-mm sieve, then dried to complete dryness in a hot air oven at 40 °C for 3 h. The dried granules were passed over a mesh of 425 μm size.

### 2.3. Determination of Bulk Density

Bulk density (g/mL) was calculated by pouring, without tapping, 50 g of each powder into a 100-mL graduated glass cylinder. The powder volume was then recorded and used to calculate the bulk density; each sample was measured in triplicate.

### 2.4. Compression of Excipients (Single, Binary, or Ternary)

Next, 150 mg of the single and binary, and 100 mg of the ternary mixtures were compressed using the Gamlen Tablet Press machine (Gamlen Tableting Ltd., Biocity Nottingham, UK). The compression forces were fixed at 100, 200, 300, 400, and 500 kg. The powders were compressed using a punch size of 6.0 mm in diameter at a speed of 60 mm/min. Force-displacement curves (work), Heckel and Kawakita analysis, hardness, and disintegration testing were all carried out upon compression of the powder samples into tablets. The test was carried out in triplicates.

### 2.5. Heckel and Kawakita Analysis of the Compression Data

A number of models are available to mathematically analyze different mechanical aspects of powder compression properties. The Heckel and Kawakita analysis still remains the most commonly used models for describing such aspects [14,15,16,17].

The Heckel equation (Equation (1)) describes the change in powder porosity as a function of the applied pressure upon powder densification.

(1) $$\ln\frac{1}{1-D}=kP+A$$

where *D* is the relative density of a powder compact at pressure P. Slope *k* is a measure of the plasticity of a compacted material. The inverse of the slope is the yield pressure of the material (*P_Y_*), which describes the tendency of the material to deform either by plastic flow or fragmentation. Accordingly, the higher the slope value, the lower the yield pressure, and the higher the ability of the material to deform plastically under pressure. Constant A is related to die filling and particle rearrangement prior to the deformation and bonding of the discrete particles.

The compression behavior of the samples can be also evaluated using Kawakita analysis of powder compression data. The Kawakita equation (Equation (2)) is used to study powder compression via the degree of volume reduction *C*.

(2) $$\frac{P}{C}=\frac{P}{a}+\frac{1}{ab}$$

where *C* is the degree of volume reduction of the powder column under the applied pressure *P*. The constant *a* is the minimum porosity of the material before compression, while the constant *b* relates to the amount of plasticity of the material. The reciprocal of *b* or *P_k_* defines the pressure required to reduce the powder bed by 50%. The *P_K_* values provide a direct indication of the strength of, or ability to resist, the granules [18,19].

### 2.6. Force-Displacement Curves

At a certain compression pressure, the volume is reduced, and the particles’ packing reaches a minimum. These changes are associated with elastic and/or plastic deformation or with brittle fracture of the particles into smaller ones. The use of force-displacement curves has been a common evaluation technique for powder compression properties. The area under the force-displacement curve is called the work of compression [5].

### 2.7. Tablets Hardness

Tablet hardness was measured using a hardness tester. Ten tablets were tested. The means and standard deviations were calculated.

### 2.8. Tablets Thickness

The thickness of the compacts (tablets’ height) and the diameter were measured using a vernier caliper in (mm). The average of 10 tablets was taken.

### 2.9. RSM Analysis

Binary mixtures were handled using the RSM technique [20] whereby preparations of binary mixtures were subjected to one-factor design using Design-Expert^®^ software version 11. Analysis was carried out using a quadratic model based on a fractional factorial design of 13 runs. The response surface plots were generated to see how the responses (yield pressure (*P_Y_*), *P_K_*, work of compression, tablet hardness) vary with an excipient mass content. Five lack-of-fit points and five replicate points were chosen for an optimal integrated variance (IV) design that provides a lower average prediction variance across the whole range of excipient mass contents. Table 1 presents the levels of the 13 runs and their corresponding mass contents.

For ternary components, a mixture design of three components was chosen in this work to find an optimal formulation. A quadratic polynomial option was chosen to model the response based on 10 runs. Similarly, five lack-of-fit points and five replicate points were chosen for optimal IV design. Table 2 presents the levels of 10 runs and their corresponding mass contents.

### 2.10. Tablet Preparation and Drug Dissolution

Two powder mixtures, each 100 g, containing 50 g metronidazole or 50 g paracetamol, and 50 g of the ternary excipient mixture (80% MCC, 15% starch, and 5% *w*/*w* Mg silicate), were blended for 10 min using an Erweka cube mixer (KB 155, Langen, Germany). The powders were compressed into tablets (1 g) containing 500 mg metronidazole or 500 mg paracetamol using a single punch machine (Manesty F3 single stroke tablet press; West Pharmaservices Ltd., Dorset, UK) with oblong punches (19 mm × 9.5 mm). Dissolution runs, sampling, and drug measurements were carried out according to the United States Pharmacopoeial-USP 31(2005) methods for each drug. For metronidazole tablets, the USP Apparatus 1 (basket) method was considered. A 0.1 N HCl solution was used as the dissolution medium. Dissolution was carried out at 37 ± 2 °C, 100 rpm rotational speed, and 900 mL of the medium. Samples (5 mL) were withdrawn at the time intervals of 5, 10, 15, 30, and 45 min, diluted, and then analyzed spectrophotometrically at 275 nm.

For paracetamol tablets, the USP Apparatus 2 (paddle) method was considered. Then, 900 mL of phosphate buffer (pH 5.8) was used as a dissolution medium. The paddle was set to rotate at 75 rpm. Samples (5 mL) were withdrawn at the time intervals of 5, 10, 15, 30, and 45 min, then diluted using phosphate buffer (pH 7.8). The samples were filtrated through a 0.45-µm glass filter and analyzed spectrophotometrically at 243 nm.

## 3. Results and Discussion

### 3.1. Results

Parameters representing the compressional behavior of a powder blend were investigated in this work with reference to the influence of a combination of plastically deforming, elastic, and brittle fracture excipients for binary and ternary mixtures. MCC, maize starch, and Mg silicate were chosen to represent the aforementioned three types of excipients, respectively. Justification for this choice was tested using Heckel and Kawakita compression analysis, whose results are shown in Table 3.

From the Heckel slope (K) order, MCC > starch > Mg silicate, the yield pressure (*Py*), which is an inverse of the Heckel slope (1/slope), was calculated. Since the lower the yield pressure, the higher the ability of the material to deform plastically under pressure, MCC is more plastically deforming than starch, which then later deforms more plastically and to a greater extent than Mg silicate. Kawakita parameters indicate that, in terms of compressibility order, or (*a*), MCC > Mg silicate > starch. Thus, MCC manifested the highest volume reduction when compressed, whereas starch manifested the lowest. This is most probably attributed to the highest bulk density recorded by maize starch [21], whereas MCC recorded the lowest. Furthermore, MCC granules were the hardest since their calculated *P_K_* value, or the force required to reduce the powder bed by *a*/2, was the highest (Table 3). In contrast, starch was the weakest granule among the three excipients.

**Figure 1:**
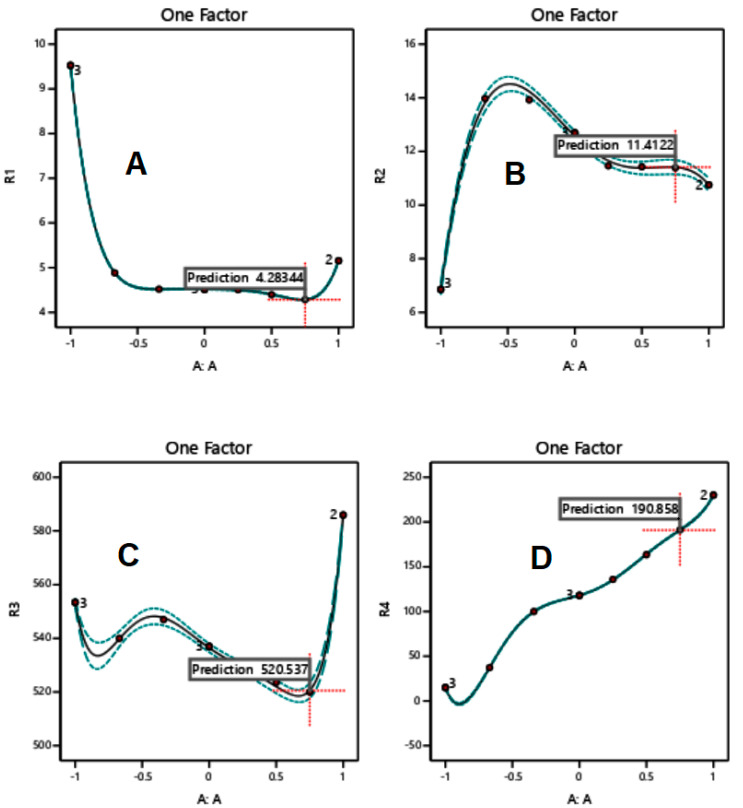
RSM of MCC-starch for a one-factor design with yield pressure (*P_Y_*) (**A**), *P_K_* (**B**), work of compression (**C**), and tablet hardness (**D**) as the responses.

**Figure 2:**
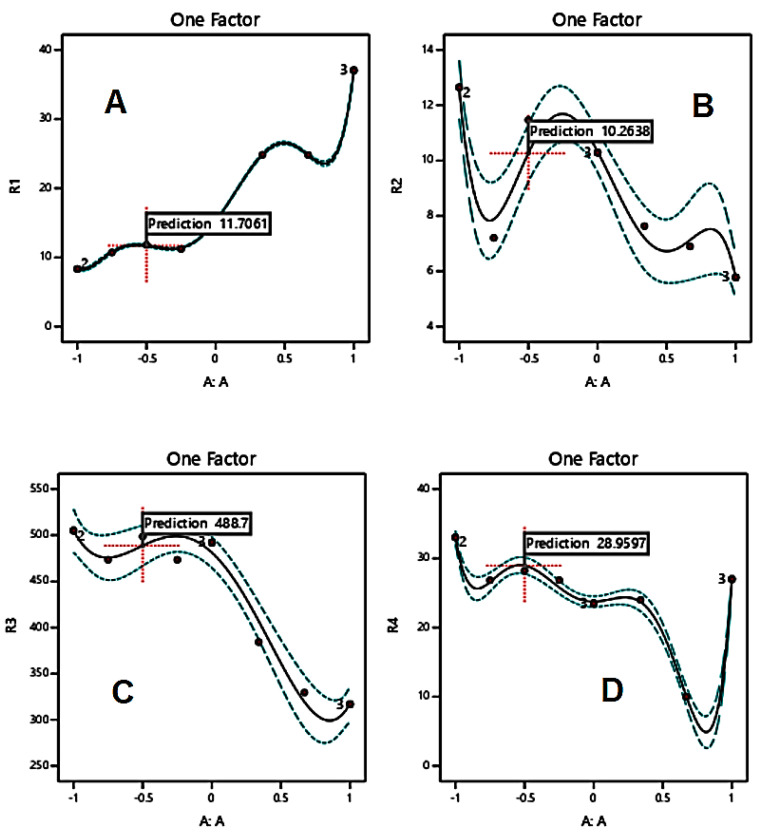
RSM of Mg silicate -starch for a one-factor design with yield pressure (*P_Y_*) (**A**), *P_K_* (**B**), work of compression (**C**), and tablet hardness (**D**) as the responses.

**Figure 3:**
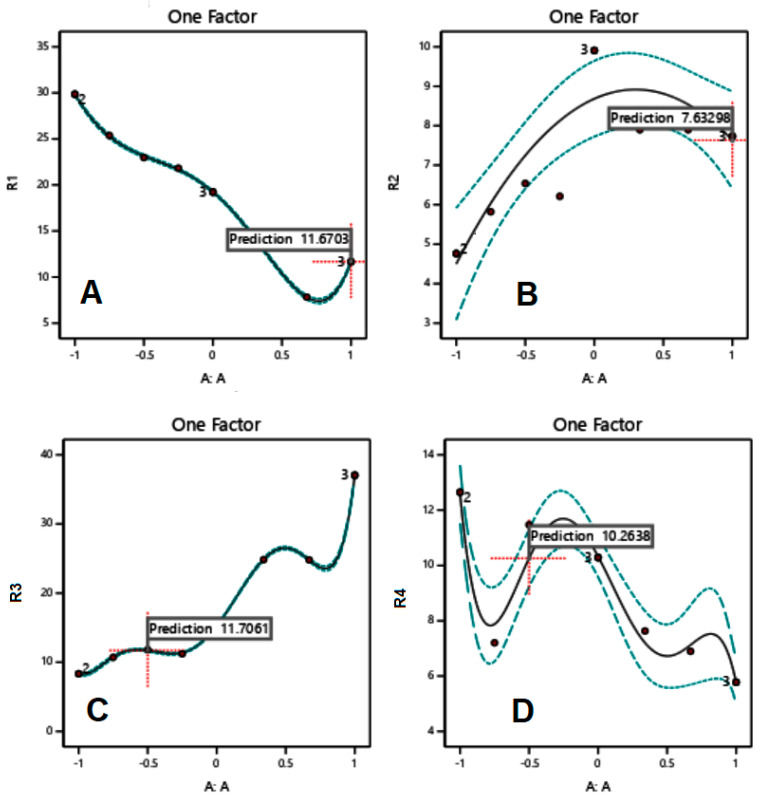
RSM of MCC-Mg silicate for a one-factor design with yield pressure (*P_Y_*) (**A**), *P_K_* (**B**), work of compression (**C**), and tablet hardness (**D**) as the responses.

When excipients were mixed according to the mass contents set by the RSM technique (Table 2), responses were recorded as presented in Figure 1, Figure 2 and Figure 3 for MCC-starch, Mg silicate-starch, and MCC-Mg silicate, respectively.

For MCC-starch binary mixtures, increasing the content of MCC lowered the value of yield pressure down to a minimum around a mass content of 80–85% *w*/*w*, increased the *P_K_* value up to a maximum around a mass content of 30% *w*/*w*, increased the compression work of mass contents of 85% *w*/*w*, and finally linearly increased the tablet hardness values. The dotted lines represent the 95% confidence band of the mean prediction at any given departure time.

For Mg silicate-starch binary mixtures, the minimum *P_Y_* value was reached at approximately 15–25% *w*/*w* mass content of Mg silicate (or 75–85% starch), the maximum *P_K_* value at approximately 25% Mg silicate or 85% starch, maximum work of compression at approximately 25–35% Mg silicate (or 65–75% starch), and the maximum hardness at approximately 15–25% *w*/*w* mass content of Mg silicate (or 75–85% starch).

Finally, for MCC-Mg silicate binary mixtures, increasing the content of MCC lowered the value of the yield pressure down to a minimum around a mass content of 80–85% *w*/*w*, increased the *P_K_* value up to a maximum around a mass content of 70–80%, linearly increased the compression work, and finally reached a maximum tablet hardness value of approximately 75–80% *w*/*w*.

**Figure 4:**
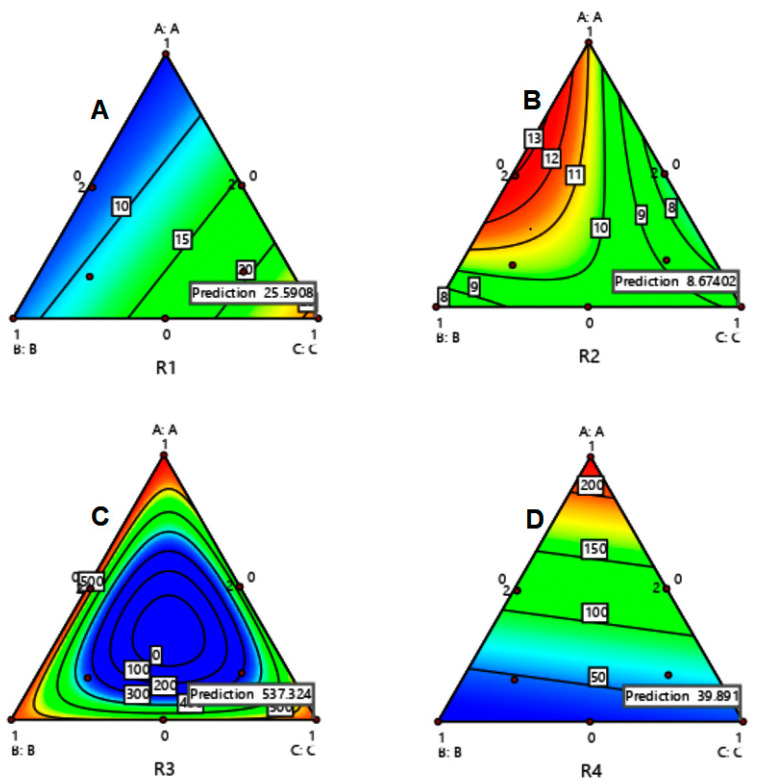
RSM of MCC-starch-Mg silicate for a three-component mixture design with yield pressure (*P_Y_*) (**A**), *P_K_* (**B**), work of compression (**C**), and tablet hardness (**D**) as the responses.

For the mixture design of the three components using RSM, the 2D contour plots are presented in Figure 4. The figure shows four plots representing yield pressure (*P_Y_*), *P_K_*, work of compression, and tablet hardness as a function of the three mixture components. Regions in red and blue colors assign high and low values, respectively, whereas the intensity of other colors designates the high and low values of excipient mass contents. In all cases, the blue and red colors present a great deal of interest in interpreting optimum composition and tableting conditions.

**Figure 5:**
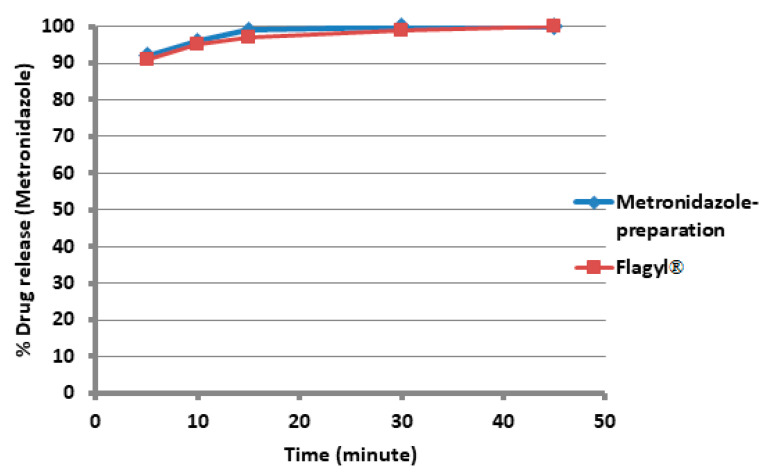
Percent drug release of metronidazole with 60% MCC, 30% starch, and 10% Mg silicate that has been prepared and Flagyl^®^. Each point is based on the average of six tablets.

**Figure 6:**
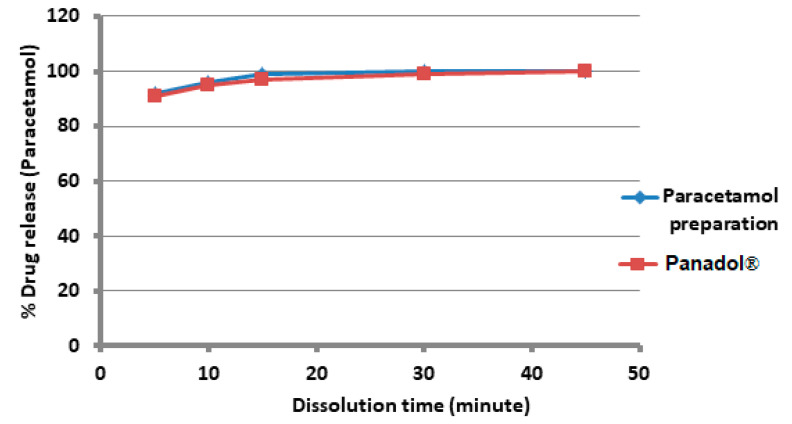
Percent drug release of paracetamol with 60% MCC, 30% starch, and 10% Mg silicate that has been prepared and Panadol^®^500 mg. Each point is based on the average of six tablets.

The ternary excipient (80% MCC, 30% starch, and 10% Mg silicate) at the contents of 10% formulated with 90% *w*/*w* metronidazole and paracetamol showed an immediate release profile, as shown in Figure 5 and Figure 6, respectively.

The two figures represent a typical example of immediate-release profiles. The tablet hardness measured was relatively high (~150 N) for both drugs. Moreover, the model drugs were almost identical to the originals of the same strengths and presented a typical immediate release profile, whereby 80% of both drugs were released at 60 min.

### 3.2. Discussion

In the investigation of the compression properties of MCC-starch mixtures, it can be noticed that binary and ternary mixtures provide better tableting behavior than individual ones. Figure 1A–C indicate that the *P_Y_*, *P_K_*, and work of compression values of individual components provide the worst compressional scenario for tableting. More specifically, MCC and starch recorded higher *P_Y_* values, lower *P_K_* values, and higher work of compression than most compositions of the MCC-starch mixtures. The foregoing, therefore, have higher plasticity, higher resistance (hardness) of the granules, and lower energy displayed upon compression, respectively. In contrast, Mg silicate-starch binary mixtures showed better tableting behavior in the individual components than their mixtures. In this regard, starch has the lowest *P_Y_* (2A), highest *P_K_* (2B), and the highest hardness (2D) of all mixtures and Mg silicate itself. Consequently, Mg silicate, as a brittle fracture material, weakens the compressional properties when added to starch. Similarly, Mg silicate, to some extent, weakens the powder/tablet properties when added to Avicel, as Figure 3 illustrates. In this regard, the addition of Mg silicate contributed to increasing the yield pressure (*P_Y_*), weakening the strength of the granules (*P_K_*), and reducing the tablet hardness. It has to be further emphasized that Mg silicate reduced the work of compression when mixed with any of the plastically/or elastically deforming materials (MCC/or starch) in Figure 2C and Figure 3C.

The role of RSM in the current work was not only employed to highlight the compression parameters of individual components and their mixtures, but was also beneficial to find the optimum mass contents for mixtures in tableting. For the plastic/elastic binary mixture, MCC/starch, it can be suggested from Figure 1A–D that an MCC mass content of approximately 80% *w*/*w* was characterized by the highest recorded plastic deformation and the lowest work of compression. Tablets created with the same composition maintained high hardness values while maintaining a reasonably high granule strength comparable to the MCC excipient when compressed alone (Figure 1B). For the brittle/elastic binary mixture, it is clear from the RSM plots of Mg silicate/starch (Figure 2A–C) that a starch/Mg silicate ratio of 75:25 or 3:1 manifested optimal compression and tableting parameters.

MCC/Mg silicate binary mixture demonstrated the dominance of MCC excipient at approximately 80% *w*/*w* mass content similar to MCC/starch. Moreover, increasing Mg silicate content with MCC or starch produced tablets with low hardness values upon increasing Mg silicate content.

It is interesting to notice that *P_K_* profiles are almost in harmony with the work of compression profiles. In other words, the increase (Figure 3B) or decrease (Figure 1B and Figure 2B) in *P_K_* values corresponds to an increase (Figure 3C) or decrease (Figure 1C and Figure 2C) in the work of compression, respectively. This is in agreement with the work of Rashid et al. [22], who stated that the two parameters were correlated. Therefore, such a finding provides a strong emphasis that the choice of a composition upon optimization of the compression of a mixture must be based on a composition that provides the maximum work of compression and concurrently the maximum Kawakita *P_K_* value.

All the aforementioned findings for binary mixtures were used to correlate compression and tableting parameters of ternary mixtures using RSM plots in Figure 4A–D. The blue regions are found to cover the position of the 80% *w*/*w* MCC mass content, representing high plastic deformation (*P_Y_*); the same material of the same content is covered in the desired red regions of Figure 4B (high granule strength), Figure 4C (high work of compression), and Figure 4D (high tablet hardness). Such MCC mass content design values are the same as those attained in binary mixtures according to RSM analysis. Since the remaining 20% is made up of starch and Mg silicate, and since the optimum starch/Mg silicate ratio is 3:1, then the optimum mass content ratio for a ternary mixture of plastic, elastic, and brittle fracture materials would be 80:15:5 for the current case. On the other hand, comparing the optimum values of the contour lines and the size of optimally colored regions in Figure 4A–D with their corresponding values in Figure 1, Figure 2 and Figure 3, it is clear that ternary mixtures perform better than binary ones.

Validation of such an optimum composition was checked using MCC: starch: Mg silicate ratios (Table 4) other than those in the RSM design, but—compared to the optimum ratio—changes were minor. The tested compositions in Table 4 confirmed the presence of an optimum mass content for a ternary mixture composed of plastic/elastic/brittle fracture materials.

When the same mixtures in Table 4 were wet granulated, all compressional properties underwent major improvement in *P_Y_*, *P_K_*, work of compression, and tablet hardness compared to the dry ternary mixtures (Table 5). The top improvement was, once again, recorded at the optimal mass content ratio of the three excipients.

The capability of the ternary mixture at the optimum ratio was well demonstrated upon formulation with the two high-strength model drugs (metronidazole and paracetamol in Figure 5 and Figure 6, respectively). The ternary mixture can, thus, be formulated through physical mixing without applying wet or dry granulation and still maintain solid, intact tablets. The dissolutions of such tablets were almost similar to their corresponding originators.

## 4. Conclusions

The optimal compressional behavior of physical mixtures comprising a combination of excipients is determined by a majority of plastic, a minority of brittle-fracture, and a moderate influence of elastic modes in the combination. The ideal performance of powder and tableting properties necessitates that all the three modes be present at the aforementioned distribution.

## Figures and Tables

**Table 1:** Levels of the 13 runs and their corresponding mass contents.

Code	−1	−0.75	−0.67	−0.5	−0.34	−0.25	0	0.25	0.34	0.5	0.67	0.75	1
**Mass content (% *w*/*w*)**	0	12.5	16.5	25	33	37.5	50	62.5	67	75	83.5	87.5	100

**Table 2:** Responses of 10 runs comprising a set of different compositions of each component (MCC, starch, Mg silicate). Responses were based on a quadratic polynomial equation.

Run	Component 1 A:A	Component 2 B:B	Component 3 C:C	Response 1 R1	Response 2 R2	Response 3 R3	Response 4 R4
1	0.17	0.15	0.66	29.85	4.76	240	73
2	0.49	0.49	0.01	4.51	12.69	537	118
3	0	1	0	9.52	6.85	553.4	15
4	0.49	0.49	0.01	4.51	12.69	537	118
5	0	0	1	19.23	9.90	529.8	35
6	0.15	0.67	0.17	12	12.69	200	26
7	0	0.50	0.49	15.29	10.29	492	23.5
8	1	0	0	5.15	10.75	585.9	230
9	0.50	0	0.49	16.085	8.31	443.5	135
10	0.50	0	0.49	16.085	8.31	443.5	135

**Table 3:** Heckel parameters for MCC, starch, and Mg silicate as single components.

	Heckel Parameters			Kawakita Parameters		
Excipients	*K*	*A*	*Py*	*a*	*Pk* = 1/*b*	Bulk Density g/cm^3^
**MCC**	0.194	0.591	5.155	0.775	10.752	0.351
**Starch**	0.105	1.123	9.524	0.591	6.854	0.510
**Mg silicate**	0.052	0.491	19.231	0.662	9.907	0.461

**Table 4:** Heckel and Kawakita parameters of ternary mixtures of composition nearby the optimum.

	Parameters				
	Heckel			Kawakita	
Mixtures MCC:Starch:Mg Silicate	K	A	*P_Y_*	*a*	*P_k_* = 1/*b*
**80:10:10**	0.027	0.498	37.037	0.589	2.001
**80:15:5**	0.043	0.527	23.256	0.606	2.795
**60:20:20**	0.025	0.384	40.000	0.618	2.617
**60:30:10**	0.034	0.502	28.347	0.611	2.701

**Table 5:** Heckel and Kawakita parameters of ternary mixtures subjected to wet granulation and of composition near the optimum.

	Parameters				
	Heckel			Kawakita	
Mixtures MCC:Starch:Mg Silicate	K	A	*P_Y_*	*a*	*P_k_* = 1/*b*
**80:10:10**	0.092	0.779	10.870	0.644	16.410
**80:15:5**	0.316	0.477	3.165	0.682	25.474
**60:20:20**	0.066	0.829	15.152	0.628	16.715
**60:30:10**	0.077	0.832	12.987	0.680	16.988

## Data Availability

The data presented in this study are available on request from the corresponding author.
